# Incorporating WGCNA and Machine Learning to Identify ADAP2 as a Critical Efferocytosis-Related Gene in Sepsis

**DOI:** 10.3390/pathogens15060596

**Published:** 2026-06-01

**Authors:** Chen Zhang, Chaozheng Xie, Zhengtao Zhang, Renjie Luo, Fang Xu

**Affiliations:** 1 Department of Critical Care Medicine, The First Affiliated Hospital of Chongqing Medical University, Chongqing 400016, China; dr_zhangchen@163.com (C.Z.);; 2 Clinical Pathology Laboratory of Pathology Diagnostic Center, Chongqing Medical University, Chongqing 400016, China

**Keywords:** sepsis, machine learning, WGCNA, ScRNA-seq, ADAP

## Abstract

Background: Sepsis, a life-threatening organ dysfunction caused by dysregulated host responses to infection, frequently involves impaired macrophage efferocytosis that leads to apoptotic cell accumulation, secondary necrosis, and persistent inflammation. Early prognostic stratification remains challenging, as current biomarkers lack sufficient specificity and sensitivity, underscoring the urgent need for novel prognosis-related indicators. Methods: We integrated bulk transcriptomic data from a discovery cohort (GSE205672) and an independent validation cohort (GSE133822) with single-cell RNA-seq profiles of early- and late-stage sepsis (GSE167363, GSE175453). WGCNA and five consensus machine-learning algorithms were combined to screen core efferocytosis-associated genes, and expression was validated via qPCR in PBMCs from sepsis patients and CLP-induced septic mice. Results: ADAP2 was identified as the core gene achieving strict consensus across all five algorithms, with early upregulation and late depletion in sepsis, predominant expression in monocytes/macrophages—particularly M1-like and IFN-responsive subsets—and a significant correlation with efferocytosis scores and immune cell infiltration. Its expression was negatively correlated with sepsis severity (SOFA score) and showed a trend toward worse survival in patients with low ADAP2 levels. Conclusions: This multi-dimensional transcriptomic study establishes ADAP2 as a candidate biomarker with potential prognostic value in sepsis, closely linked to macrophage efferocytosis. These findings may aid early risk stratification and inform macrophage-directed immunotherapies, although prospective validation and functional studies are required.

## 1. Introduction

Sepsis is defined as life-threatening organ dysfunction resulting from a dysregulated host response to infection, which leads to high mortality [1]. The substantial global burden of sepsis was highlighted by the 2017 estimates of 49 million cases and 11 million related deaths, which represented nearly 20% of all deaths worldwide [2,3]. A primary cause of mortality in critically ill patients is sepsis-induced acute lung injury [4], a condition characterized by uncontrolled pulmonary inflammation and excessive immune cell infiltration [5]. Despite progress in critical care, effective targeted therapies and specific biomarkers for sepsis remain elusive [6,7,8]. Thus, a deeper understanding of the immunoregulatory mechanisms governing sepsis is urgently needed to improve clinical outcomes.

In the inflammatory response, many immune cells, such as neutrophils, undergo apoptosis. If these apoptotic cells are not promptly cleared, they undergo secondary necrosis, releasing damage-associated molecular patterns (DAMPs), such as high mobility group box 1 (HMGB1), which further exacerbates inflammation [9]. Macrophages play a central role in the immunoregulation of sepsis [10,11]. Under physiological conditions, macrophages clear apoptotic cells through efferocytosis, promoting inflammation resolution and tissue repair [10,12]. This process involves specific receptors that recognize apoptotic cells, activate downstream signaling pathways, and drive macrophage polarization toward an anti-inflammatory M2 phenotype [4]. Impaired efferocytosis is closely associated with the progression of sepsis pathology [13]. Studies indicate that impaired efferocytosis leads to the accumulation of apoptotic cells and secondary necrosis, releasing pro-inflammatory mediators that exacerbate pulmonary inflammation [4,14]. Key receptors such as MerTK, when downregulated, inhibit macrophage phagocytic activity and impair inflammation resolution [15]. This damage further weakens the macrophage transition from pro-inflammatory M1 to repair-oriented M2 phenotypes, forming a vicious cycle [4]. These findings suggest that macrophage efferocytosis-related molecules may serve as a key target for sepsis intervention.

Interferon-stimulated genes (ISGs) are well-recognized mediators of the host response to sepsis, with members such as IFITs, MX1, and OAS proteins characterized as antiviral effectors or prognostic biomarkers [16,17]. Whether ISGs also participate in non-antiviral immunoregulatory processes, particularly macrophage efferocytosis, critical for inflammation resolution, remains largely unexplored. Notably, ISG15 was recently identified as a key regulator of efferocytosis via mitochondrial remodeling [18], suggesting broader immunoregulatory functions of ISGs beyond antiviral defense. ADP-ribosylation factor GTPase-activating protein (ArfGAP) with dual PH domains 2 (ADAP2) is an ISG whose primary function in antiviral innate immune responses has been elucidated [19,20,21]. Notably, ADAP2 contains an ArfGAP domain [21]. Arf family GTPases, such as Arf6, are key molecular switches regulating intracellular vesicle trafficking, membrane remodeling, and endocytosis [22,23], functions that are mechanistically distinct from the canonical antiviral roles of most ISGs. Nevertheless, the potential involvement of ADAP2 in efferocytosis and its clinical relevance in sepsis have not been investigated.

In recent years, high-throughput omics technologies have been widely utilized to identify transcriptomic biomarkers for sepsis [16,24]. However, traditional bioinformatic studies relying on simple differential expression across single datasets frequently exhibit limited cross-cohort reproducibility due to the profound clinical heterogeneity of sepsis. To address these analytical bottlenecks, the integration of weighted gene co-expression network analysis (WGCNA) with machine learning algorithms provides a rigorous computational framework. WGCNA systematically clusters functionally related genes, while ensemble machine learning models strictly filter out cohort-specific noise, thereby enabling the extraction of highly conserved and robust predictive features from complex immune landscapes [25].

In this study, we focused on the role of macrophage efferocytosis-related genes in sepsis. By integrating weighted WGCNA with multiple machine learning algorithms, we screened for key genes associated with efferocytosis in sepsis in peripheral blood mononuclear cells (PBMCs). Through the analysis of multi-dimensional transcriptomic data and experimental validation, we aimed to address the following issues: (1) the expression characteristics and clinical associations of efferocytosis-related genes in sepsis; (2) the specific immunophenotypic associations between efferocytosis-related genes and the sepsis microenvironment, particularly regarding macrophage subsets. The findings may provide new insights into molecular subtyping and targeted therapies for sepsis.

## 2. Materials and Methods

### 2.1. Gene Expression Profile Collection

RNA sequencing (RNA-seq) data were collected from the gene expression profile GSE205672 (161 sepsis and 299 healthy samples) and GSE133822 (20 healthy, 14 early sepsis and 16 late sepsis samples), downloaded from the Gene Expression Omnibus (GEO) database. GSE205672 was used as the primary discovery cohort; GSE133822 served as an independent validation dataset and was not merged for model training. For single-cell RNA sequencing (scRNA-seq) analyses, the datasets GSE175453 and GSE167363 were integrated, collectively providing PBMC transcriptomes from 4 early sepsis patients, 4 late sepsis patients, and 4 healthy individuals as controls. Detailed sample information is provided in Supplementary Table S1.

### 2.2. Identification of Differentially Expressed Genes

For the GSE205672 dataset, duplicate genes were collapsed by retaining the highest mean expression, genes with zero counts across all samples were removed, and expression values were log_2_(FPKM + 1)-transformed. The R package limma [26] (version 3.60.4) was used to identify the differentially expressed genes (DEGs) between healthy and sepsis patients and between the high and low efferocytosis score groups. |log_2_FC| ≥ 1 and a Benjamini–Hochberg adjusted *p* < 0.05 were considered significant.

### 2.3. Efferocytosis-Related Gene Score

Genes with efferocytosis signatures (Supplementary Table S2) were acquired from the Kyoto Encyclopedia of Genes and Genomes (KEGG, https://www.kegg.jp/entry/hsa04148, accessed on 23 June 2025), and the efferocytosis status of sepsis was assessed using the single-sample Gene Set Enrichment Analysis (ssGSEA) algorithm in the R package GSVA [27] (version 1.48.3) for the GSE205672 dataset. The patients were divided into high and low efferocytosis score groups based on the efferocytosis score.

### 2.4. Functional Enrichment Analysis

The DEGs were subjected to enrichment analysis with Gene Ontology (GO), KEGG terms and Gene Set Enrichment Analysis (GSEA) using the R package clusterProfiler [28] (version 4.8.3) to obtain gene set enrichment results.

### 2.5. Weighted Gene Co-Expression Network Analysis (WGCNA) and Candidate Gene Identification

First, we excluded the 50% of genes with the lowest median absolute deviation (MAD) from the expression matrix. Using the goodSamplesGenes function in the R package WGCNA [25,29] (version 1.73), we removed outlier genes and samples, discarding 11 sepsis and 6 healthy control samples that were clearly aberrant. The optimal soft-thresholding power was determined using the pickSoftThreshold function. A power of β = 16 was selected, at which the scale-free topology fit index (R^2^) reached 0.89 and the mean connectivity stabilized (Supplementary Figure S1A), satisfying the approximate scale-free topology criterion while preserving sufficient gene connectivity. Subsequently, hierarchical clustering was applied to identify feature modules, with a minimum module size of 30 genes. Modules with a dissimilarity less than 0.25 were merged, yielding a total of 13 co-expression modules. The gray module comprised genes that could not be assigned to any module.

Finally, we assessed the correlation between clinical traits and gene expression. Genes with robust co-expression within the clinically significant modules were identified based on the WGCNA criteria: absolute Module Membership (|MM|) > 0.8 and absolute Gene Significance (|GS|) > 0.2. These genes were designated as highly connected module genes and were subsequently utilized as candidate features for downstream analysis.

### 2.6. Identification of Key Genes Using Machine Learning

To identify robust biomarkers for distinguishing sepsis status, we performed comprehensive feature selection integrating five distinct machine learning algorithms (Lasso, GlmBoost, RF, Stepwise both, Stepwise back). Gene expression data from 443 samples encompassing 105 genes were preprocessed via z-score normalization. The dataset was organized with DiseaseStatus as the binary outcome variable (Sepsis vs. Healthy). To systematically minimize overfitting during model training, hyperparameter tuning and feature selection for each algorithm were conducted using 10-fold internal cross-validation. All analyses were conducted in R version 4.4.1 with the caret package [30] (version 6.0-94), and a fixed random seed (100) was set to ensure reproducibility. Consensus features shared across all five algorithms were designated as core biomarkers, and their distributions were visualized using UpSet plots to depict method-specific gene selections and their overlaps. The cross-validated classification performance (AUC, sensitivity, and specificity) of each algorithm is summarized in Supplementary Table S3.

To prevent the exclusion of features with high biological relevance but model-specific importance, we adopted an ensemble voting (soft consensus) approach. All genes selected by at least one algorithm were cataloged and classified into three confidence tiers based on selection frequency: Strict Consensus (selected by 5/5 methods), High Confidence (3-4/5), and Algorithm-Specific (1-2/5). The full tiered list with per-method importance metrics is provided in Supplementary Table S4.

### 2.7. Immune Infiltration Analysis

We utilized Cell-type identification by estimating relative subsets of RNA tran-scripts (CIBERSORT) to quantify the abundances of infiltrating immune cells with the LM22 signature matrix, which encompasses 22 distinct immune cell types. Following the removal of genes with zero expression across all 443 samples (150 sepsis patients and 293 healthy controls), deconvolution analysis was performed with quantile normalization applied. Differential immune cell infiltration patterns between the sepsis and healthy groups were assessed using the Mann–Whitney U test and visualized using boxplots and heatmaps. Spearman’s rank correlation analysis was conducted to evaluate associations between core genes and immune cell proportions, with a false discovery rate (FDR) correction applied to account for multiple testing.

### 2.8. ScRNA-Seq Data Analysis

We performed scRNA-seq analysis using the Seurat [31] package (version 5.0.0). After quality control to exclude cells with mitochondrial gene content > 15% or gene counts < 200 or >3000, we normalized the data using SCTransform. To address batch effects across different sequencing cohorts, we performed data integration using the reciprocal PCA (RPCA) approach within the Seurat integration pipeline. Dimensionality reduction was performed using principal component analysis (PCA) followed by UMAP and t-SNE visualization based on the top 30 principal components. Cell clustering was conducted at a resolution of 0.5, and cell identities were annotated by comparing marker gene expression patterns with established immune cell signatures. We identified 10 major cell types including T cells, B cells, plasma cells, NK/NKT cells, monocytes/macrophages, dendritic cells, neutrophils, platelets, and erythroid precursor cells. For deeper analysis, we extracted the monocyte/macrophage population for subclustering. Cell–cell communication networks were investigated using CellChat (version 1.6.1) with the human Secreted Signaling and Cell–Cell contact database.

Trajectory analysis of the inflammatory maturation axis (classical monocytes, M1, and IFN-responsive macrophages) was performed using the Slingshot R package (version 2.8.0). To prevent topological artifacts, trajectories were inferred independently for each clinical stage using UMAP embeddings, anchoring the root at classical monocytes. Pseudotime values were min-max normalized (0–100%) to represent relative progression. To mitigate scRNA-seq zero-inflation, the normalized pseudotime was divided into ten equal bins, and the percentage of ADAP2-expressing (ADAP2+) cells within each bin was calculated to compare dynamic trends across stages.

### 2.9. RNA Extraction and Quantitative Reverse Transcription Polymerase Chain Reaction (qRT-PCR)

Total RNA was extracted from PBMC samples of septic patients (*n* = 22) and healthy individuals (*n* = 11) using TRIzol reagent (Cat. No. 15596026CN, Invitrogen, Waltham, MA, USA) following the manufacturer’s instructions. Subsequently, complementary DNA (cDNA) was synthesized via reverse transcription PCR (RT-PCR) using the PrimeScript^TM^ RT Kit (Cat. No. RR037A, Takara, Shiga, Japan). Transcript levels of target genes were quantified via SYBR Green-based real-time quantitative PCR (qPCR). Primer sequences for target gene amplification are provided in Supplementary Table S5. Relative gene expression levels were calculated using the 2^−ΔΔCt^ method, with glyceraldehyde-3-phosphate dehydrogenase (GAPDH) serving as the internal reference gene.

### 2.10. Cecum Ligation and Puncture (CLP)-Induced Sepsis Mice Model

Male C57BL/6 mice (8 weeks old, 20–25 g) were purchased from the Animal Experiment Center of Chongqing Medical University (Chongqing, China), acclimatized for 1 week, and randomly allocated to sham or CLP groups (*n* = 5/group); following 12 h fasting (with free water access), the mice were anesthetized with intraperitoneal 1% sodium pentobarbital (50 mg/kg; Cat. No. P3761, Sigma-Aldrich, St. Louis, MO, USA), a 1 cm midline abdominal incision was made after shaving and disinfection with povidone iodine, the cecum was exteriorized, 75% of its distal length was ligated with 3-0 non-absorbable sutures, punctured once with a 21-gauge sterile needle (with gentle extrusion of intestinal contents), and repositioned, and the incision was closed in layers, followed by subcutaneous fluid resuscitation with pre-warmed (37 °C) sterile 0.9% NaCl (1 mL/10 g body weight), while sham mice underwent laparotomy without ligation/puncture. All experiments were approved by the Animal Ethics Committee of Chongqing Medical University.

### 2.11. Statistical Analysis

Quantitative data are presented as the mean ± standard deviation (SD). The normality of the data distribution was assessed using the Shapiro–Wilk test. For comparisons between two groups, an unpaired Student’s *t*-test was employed for normally distributed data, while the Mann–Whitney U test was used for non-parametric data. For multiple-group comparisons, one-way ANOVA followed by Tukey’s HSD post hoc test (parametric) or the Kruskal–Wallis test (non-parametric) was performed. For high-throughput transcriptomic analyses, *p*-values were adjusted for multiple testing using the Benjamini–Hochberg false discovery rate (FDR) method. Statistical significance was defined as two-tailed *p* < 0.05. All analyses were performed using R software (version 4.4.1).

## 3. Results

### 3.1. Identification of Differentially Expressed Genes in Sepsis

**Figure 1:**
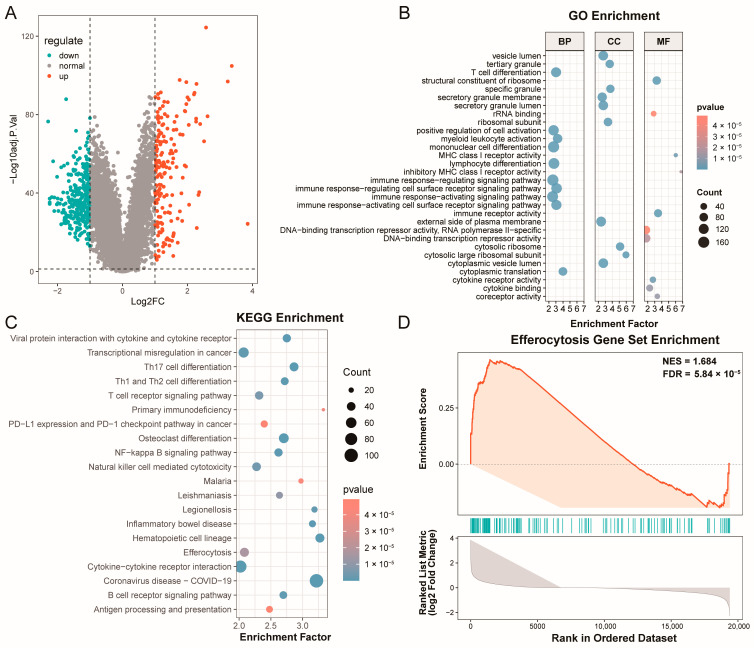
Functional enrichment of differentially expressed genes (DEGs). (**A**) Volcano plot of DEGs from differential analysis of peripheral blood mononuclear cells (PBMCs) and monocytes (161 sepsis patients, 299 healthy controls); here, 907 upregulated (red) and 1757 downregulated (teal) DEGs are shown. Differential expression was determined using the limma package with Benjamini–Hochberg FDR correction. (**B**) Gene Ontology (GO) enrichment dot plot (biological processes [BP], cellular components [CC], molecular functions [MF]); dots represent enriched terms (size = gene count; color = *p*-value). (**C**) Kyoto Encyclopedia of Genes and Genomes (KEGG) pathway enrichment dot plot (size = gene count; color = *p*-value). (**D**) GSEA enrichment analysis of the efferocytosis gene set. Genes are ranked in descending order by log_2_FC of sepsis samples versus healthy controls (Upper panel: Dynamic enrichment score curve; Middle panel: Positions of gene set members in the ranked gene list; Lower panel: Distribution of log_2_FC values for all ranked genes). Normalized enrichment score (NES) and false discovery rate (FDR) were calculated to assess the enrichment significance.

To investigate the functional characteristics of DEGs, we performed differential analysis on PBMCs and monocytes from 161 sepsis patients and 299 healthy controls, identifying 907 upregulated and 1757 downregulated DEGs (Figure 1A). GO enrichment analysis (Figure 1B) revealed significant terms in biological processes (BP), cellular components (CC) and molecular functions (MF) such as “vesicle lumen”, “tertiary granule”, “T cell differentiation” and “structural constituent of ribosome”. KEGG pathway analysis (Figure 1C) identified key enriched pathways, including “Coronavirus disease–COVID-19”; “Th17 cell differentiation”; “Cytokine–cytokine receptor interaction”; and notably “Efferocytosis” (*p* = 1.995 × 10^−5^), indicating active clearance of apoptotic cells and its potential role in modulating immune responses during infection. Further GSEA revealed that the efferocytosis gene set was significantly enriched in sepsis (NES = 1.684, FDR = 5.84 × 10^−6^) (Figure 1D).

### 3.2. Identification of Key Modules Using WGCNA

**Figure 2:**
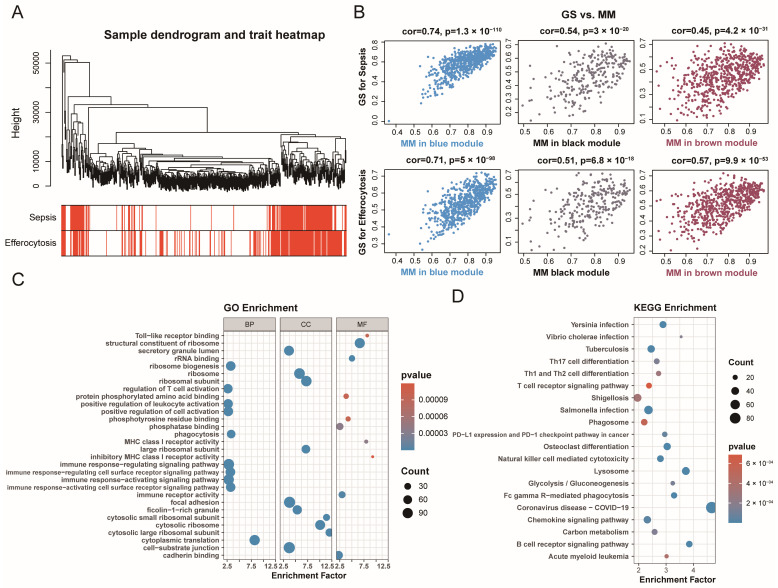
WGCNA-based co-expression module analysis in sepsis. (**A**) Sample dendrogram (**top**) and trait heatmap (**bottom**) from Weighted Gene Co-expression Network Analysis (WGCNA) of the GSE205672 dataset (460 total samples; 17 outliers excluded). The heatmap links samples to traits (“Sepsis”, “Efferocytosis”). (**B**) Scatter plots of Module Membership (MM) vs. Gene Significance (GS) for representative modules (blue, black, brown); genes were filtered using |MM| > 0.8 and |GS| > 0.2 (x-axis: MM; y-axis: GS for Sepsis/Efferocytosis). (**C**) GO enrichment dot plot; dot size = gene count; dot color = *p*-value (darker = more significant). (**D**) KEGG pathway enrichment dot plot; dot size = gene count; dot color = *p*-value (darker = more significant). Key enriched pathways include T-cell-related immune regulation and phagocytosis.

To identify gene modules critically associated with sepsis-induced efferocytosis, we performed WGCNA using the GSE205672 dataset with 460 samples. Initially, a dendrogram based on Euclidean distance was constructed, and 17 outlier samples were excluded (Figure 2A). Subsequently, a gene co-expression network was built using a soft threshold β = 16 (Figure S1A), and the Topological Overlap Matrix (TOM) was hierarchically clustered (Figure S1D). The Dynamic Tree Cut algorithm identified 13 co-expression modules (Figure S1B). To identify modules most relevant to sepsis and efferocytosis, correlation analysis was performed. Figure S1C shows the correlation between modules and traits. The blue module exhibited the strongest positive correlation with sepsis and efferocytosis (r = 0.72, *p* = 4 × 10^−82^; r = 0.7, *p* = 2 × 10^−6^). The black and brown modules also showed significant associations with sepsis and efferocytosis.

We further selected 654 genes with |MM| > 0.8 and |GS| > 0.2 from the modules (Figure 2B). To explore the functional roles of these highly associated modules, GO and KEGG enrichment analyses (Figure 2C,D) were conducted. The results revealed significant enrichment in T-cell-related immune regulation, ribosome, and phagocytosis, indicating important roles in immune regulation and cellular uptake.

### 3.3. Machine Learning Identifies Key Efferocytosis-Related Genes in Sepsis

**Figure 3:**
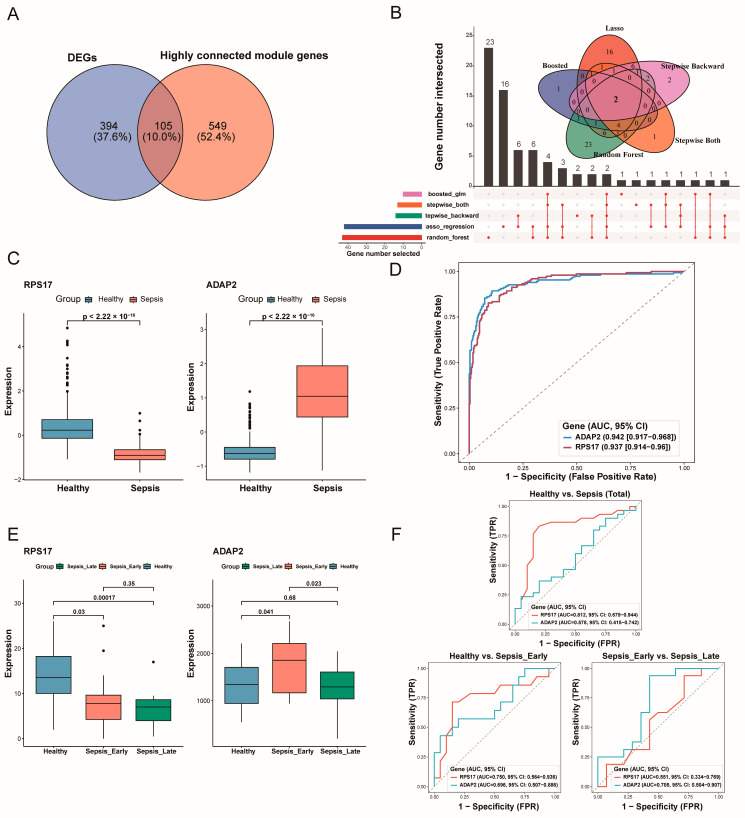
Screening and validation of core genes linked to sepsis efferocytosis via module-gene analysis and machine learning. (**A**) Venn diagram showing the intersection between 654 module-associated genes and 499 DEGs; here, 105 common genes are identified. (**B**) Top: Venn diagram of core genes screened by five machine learning methods (intersection yields ADAP2 and RPS17). Bottom: UpSet plot displaying the number of genes identified by each machine learning method. (**C**) Box plots of ADAP2 and RPS17 expression in control vs. sepsis groups (dots = individual samples). (**D**) Receiver operating characteristic (ROC) curve (AUC) assessing the predictive value of ADAP2 and RPS17 for sepsis. Validation of candidate features in the GSE133822. (**E**) Expression profiles of ADAP2 and RPS17 across healthy, early sepsis, and late sepsis groups. (**F**) ROC curves assessing the AUC of ADAP2 and RPS17 across distinct clinical state comparisons.

These 654 genes were then intersected with 499 DEGs identified via differential expression analysis, resulting in 105 common genes (Figure 3A). To identify the key genes, we applied five machine learning methods to screen for critical genes (Figure 3B and Figure S3). The intersection of these methods identified two core genes associated with sepsis efferocytosis: ADAP2 and RPS17 (Figure 3B and Figure S3C). While ADAP2 and RPS17 were the only genes selected by all five methods, eight additional genes (e.g., CTSD, PLBD1, IFNGR2) were chosen by three or four methods and ranked among the top features by random forest importance (Figure S2; Supplementary Table S4). These high-confidence genes represent promising candidates for future investigation and are reported here to avoid undue loss of biological information. AUC curves and differential expression analysis indicated that ADAP2 (AUC = 0.942, 95% CI 0.917–0.968) and RPS17 (AUC = 0.937, 95% CI 0.914–0.96) could serve as predictive markers for sepsis (Figure 3C,D). To validate the reliability of these findings, we analyzed ADAP2 and RPS17 in the independent GSE133822 dataset (Figure 3E,F). Compared with the controls, RPS17 expression was significantly lower in sepsis, consistent with previous results. Notably, ADAP2 expression was initially upregulated in early sepsis but decreased in late sepsis, showing a trend of depletion. Furthermore, ROC analysis indicated that ADAP2 demonstrated greater discriminatory value for stratifying early versus late-stage sepsis (AUC = 0.705) (Figure 3F).

### 3.4. The Relationship Between Key Genes and Immune Cell Infiltration

**Figure 4:**
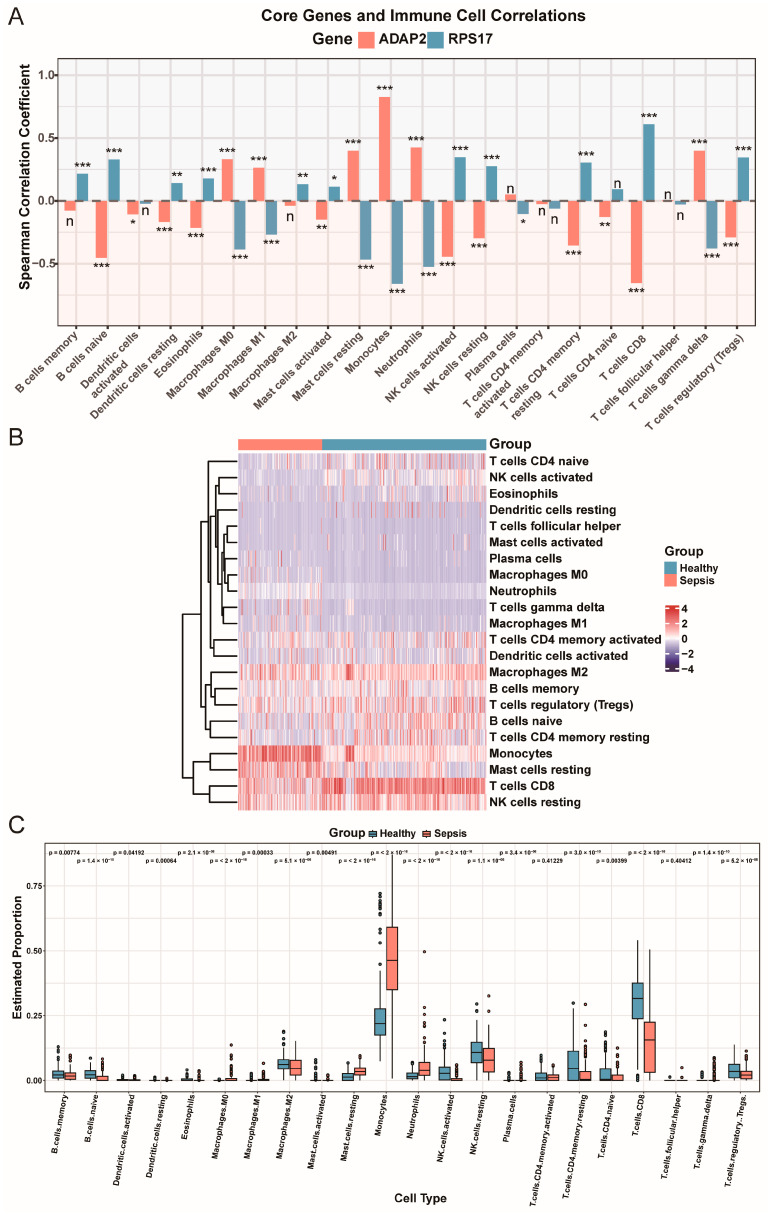
Immune cell infiltration profiles and their correlations with core genes (ADAP2, RPS17) in sepsis. (**A**) Spearman correlation plot of core genes (ADAP2: orange; RPS17: blue) with 22 immune cell types (identified from the GSE205672 dataset); n, not significant (*p* ≥ 0.05), * *p*  <  0.05, ** *p*  <  0.01, *** *p*  <  0.001. The *p*-values were calculated using the Kruskal–Wallis test. (**B**) Heatmap of 22 immune cell infiltration abundances in Healthy vs. Sepsis groups (GSE205672 dataset); color intensity reflects infiltration level (red = higher abundance; blue = lower abundance). (**C**) Box plots depicting ADAP2 (orange) and RPS17 (blue) expression levels across different immune cell types, stratified by Healthy (blue boxes) and Sepsis (red boxes) groups.

In sepsis patients, immune cell infiltration differs significantly from that in healthy individuals. To investigate the immune cell profile in sepsis, the GSE205672 dataset was used to identify 22 immune cell types. The main immune cells infiltrating in the sepsis and control samples were monocytes, NK cells, CD8+ T cells, and M2 macrophages (Figure 4A,B). The correlation between these immune cells and the target genes was examined. Figure 4C shows that ADAP2 was significantly positively correlated with monocytes (r = 0.83, *p* = 1 × 10^−112^) and M1 macrophages (r = 0.26, *p* = 1.5 × 10^−8^), while RPS17 was significantly negatively correlated with monocytes (r = −0.66, *p* = 1.9 × 10^−55^) and M1 macrophages (r = −0.27, *p* = 9.7 × 10^−9^). These findings indicate a close relationship between immune cells (particularly monocyte-macrophages) and ADAP2 and RPS17 in the inflammatory response of sepsis.

While this strong positive correlation (r = 0.83) highlights the co-occurrence of ADAP2 and monocytes, we recognized that such bulk-level metrics might heavily reflect a cell composition bias—driven by the lineage-specific expression of ADAP2—rather than direct functional interactions. Therefore, scRNA-seq was subsequently utilized to decouple this compositional effect and investigate the intrinsic state of ADAP2-expressing cells.

### 3.5. Single-Cell Analysis of Early-Stage and Late-Stage Sepsis

**Figure 5:**
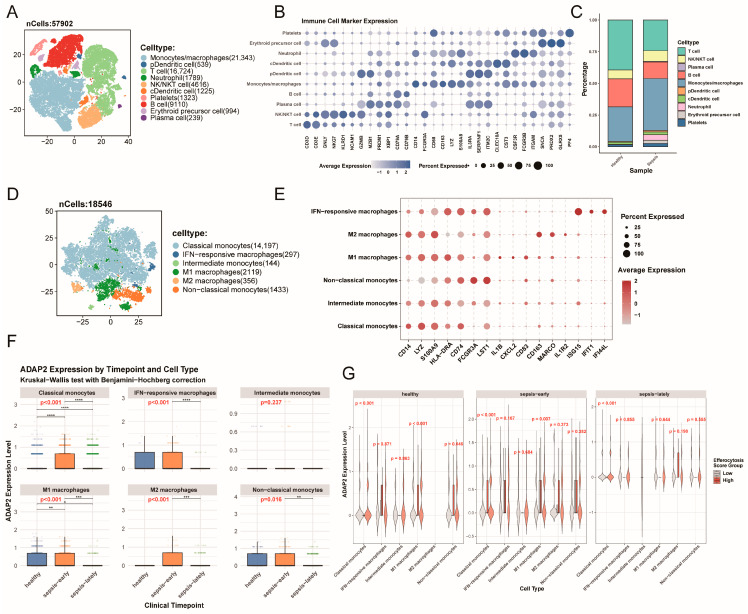
Single-cell RNA-seq analysis of ADAP2 expression and efferocytic activity in immune cells across sepsis stages. (**A**) UMAP plot of 57,902 quality-controlled PBMCs (from GSE175453 and GSE167363 datasets) clustered into 10 immune cell types (annotated on the plot). (**B**) Dotplot of canonical marker gene expression (average expression: blue gradient; percent expression: dot size) across clustered cell types, validating cell subset annotation. (**C**) Stacked bar plot showing the proportion of each immune cell type in Healthy, Early Sepsis, and Late-Sepsis groups. (**D**) UMAP visualization detailing the subclustering of monocyte and macrophage populations. (**E**) Dot plot illustrating the expression profiles of canonical marker genes utilized for the precise identification of distinct monocyte and macrophage subsets. (**F**) Box plots of ADAP2 expression in monocyte/macrophage subsets across clinical stages (Kruskal–Wallis test with Benjamini–Hochberg correction); * *p*  <  0.05, ** *p*  <  0.01, *** *p*  <  0.001, **** *p*  <  0.0001. (**G**) Correlation plot of ADAP2 expression and efferocytosis score in monocyte/macrophage subsets.

To investigate the inflammatory cell responses at different stages of sepsis, we analyzed PBMC single-cell RNA-seq data from healthy controls, early-stage, and late-stage sepsis patients (datasets GSE175453 and GSE167363). After quality control, 57,902 cells were retained and clustered into ten cell types: T cells, NK/NKT cells, plasma cells, B cells, monocytes/macrophages, pDCs, cDCs, neutrophils, and platelets (Figure 5A–C and Figure S3A–C). The expression patterns of canonical marker genes confirmed the accurate annotation of each subpopulation (Figure 5B). We then examined the expression of ADAP2 and RPS17 across the cell types. RPS17, a ribosomal gene, showed consistent expression in all immune cells without notable heterogeneity, whereas ADAP2 was predominantly expressed in monocyte/macrophage (Figure S3E).

Monocytes and macrophages play pivotal roles in the immune response to sepsis and are essential for its immunoregulation [32]. In this study, we identified subpopulations of monocytes/macrophages. Based on characteristic expression profiles, seven distinct subsets were defined: non-classical monocytes, classical monocytes, intermediate monocytes, IFN-responsive macrophages, M1 macrophages, and M2 macrophages (Figure S3D and Figure 5D,E). ADAP2 exhibited the same expression pattern as observed in the bulk transcriptomic data, with a significant decline in M1 and IFN-responsive macrophages during late-stage sepsis (*p* < 0.001) (Figure 5F). Using a set of efferocytosis-related genes (please see Table S2), we scored each cell to assess the changes in the efferocytic activity of monocyte/macrophage subsets across sepsis stages (Figure S3F). The analysis revealed that efferocytosis was most pronounced in early-stage sepsis within M1 macrophages and IFN-responsive macrophages. Moreover, ADAP2 expression in M1 macrophages from patients with early-stage sepsis overlapped with cells exhibiting high efferocytosis score (Figures S3G and Figure 5G). To evaluate the association between ADAP2 expression and macrophage efferocytic function, we stratified macrophages based on ADAP2 positivity. Notably, key efferocytosis-associated genes (e.g., CALR, SIGLEC10, CX3CR1, C1QA, and ADAM10) were markedly upregulated in ADAP2+ macrophages (Figure S3H).

To investigate the temporal dynamics of ADAP2 expression, we performed pseudotime trajectory analysis on the pro-inflammatory and IFN-responsive macrophage lineage (Figure S4A,B). Along the normalized differentiation axis, the proportion of ADAP2+ cells exhibited a robust induction during early-stage sepsis. In contrast, this upregulation was markedly blunted in late-stage sepsis, with ADAP2 expression declining toward the terminal stages of the trajectory (Figure S4C). These dynamic trends suggest that ADAP2 induction is primarily associated with the early inflammatory phase of sepsis and becomes attenuated as the disease progresses.

### 3.6. Interactions Between Macrophages, T Cells and Neutrophils in Sepsis

**Figure 6:**
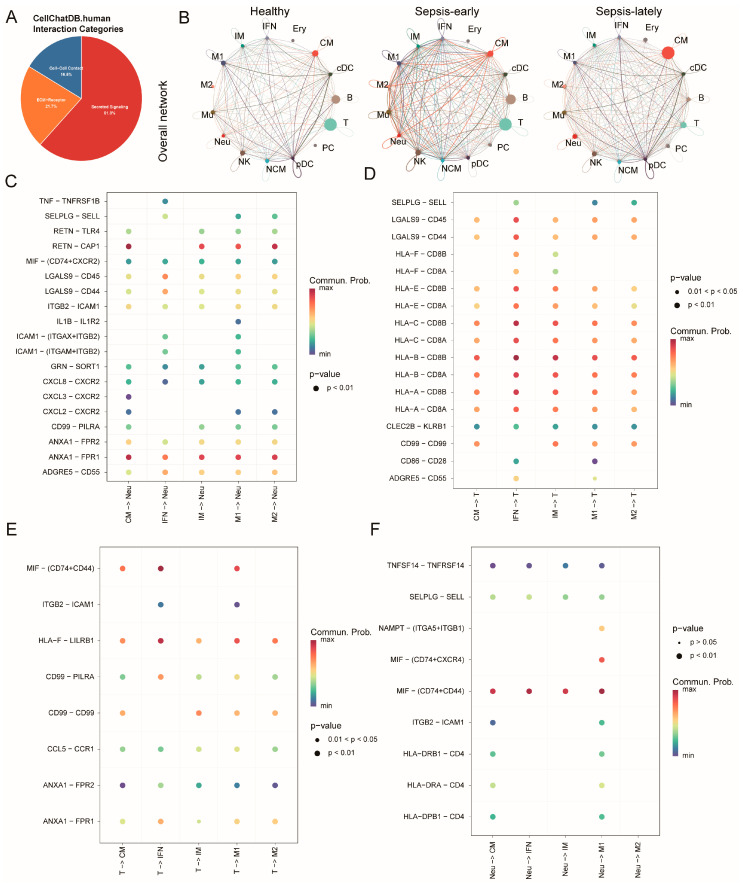
CellChat analysis of immune cell intercellular communication across sepsis stages. (**A**) Pie chart of intercellular interaction categories from CellChatDB.human, including Secreted signaling and Cell–cell contact. (**B**) Overall intercellular communication network visualizations for Healthy, Early Sepsis, and Late-Sepsis groups; nodes represent immune cell types, edges represent communication links. Abbreviations: B, B cell; cDC, conventional dendritic cell; CM, classical monocytes; Ery, erythroid precursor cell; IFN, IFN-responsive macrophages; IM, intermediate monocytes; M1, M1 macrophages; M2, M2 macrophages; Mu, monocytes/macrophages (unsubtyped); NCM, non-classical monocytes; Neu, neutrophil; NK, NK/NKT cell; PC, plasma cell; pDC, plasmacytoid dendritic cell; T, T cell. (**C**–**F**) Ligand-receptor (L-R) interactions between immune subsets in the Early Sepsis group. Bubble plots display directional communication networks: (**C**) macrophages to neutrophils, (**D**) macrophages to T cells, (**E**) T cells to macrophages, and (**F**) neutrophils to macrophages. The x-axis indicates specific sender-to-receiver pairs, and the y-axis lists the corresponding L-R pairs. Dot color represents communication probability (Commun. Prob.); dot size indicates statistical significance (*p*-values).

To characterize the immune cell intercellular communication in sepsis, CellChat analysis was performed using single-cell RNA sequencing data, incorporating Secreted signaling and Cell–cell contact from CellChatDB.human (Figure 6A). The overall communication network was most dense in early sepsis, significantly higher than in healthy controls and late sepsis (Figure 6B). Furthermore, macrophage-related signals were the most enriched across cell clusters (Figure 6B). Subsequently, further analysis revealed stage-specific crosstalk patterns (Figure S5A,B): macrophage–neutrophil interactions dominated in early sepsis, while macrophage-T cell communication became prominent in late sepsis. Pathway enrichment indicated that these putative interactions were statistically associated with gene sets related to anti-inflammatory and efferocytosis functions (Figure S5C). Key putative ligand–receptor pairs were predicted: the ANXAl–FPR1 axis was inferred to participate in macrophage-neutrophil communication, while LGALS9-CD44 and LGALS9-CD45 were mathematically enriched in macrophage-T cell crosstalk (Figure 6C,D). Additionally, reverse regulatory signals from neutrophils and T cells to macrophages were detected, reflecting a bidirectional immune regulatory network (Figure 6E,F).

### 3.7. Expression of ADAP2 and Efferocytosis-Related Genes in Sepsis

**Figure 7:**
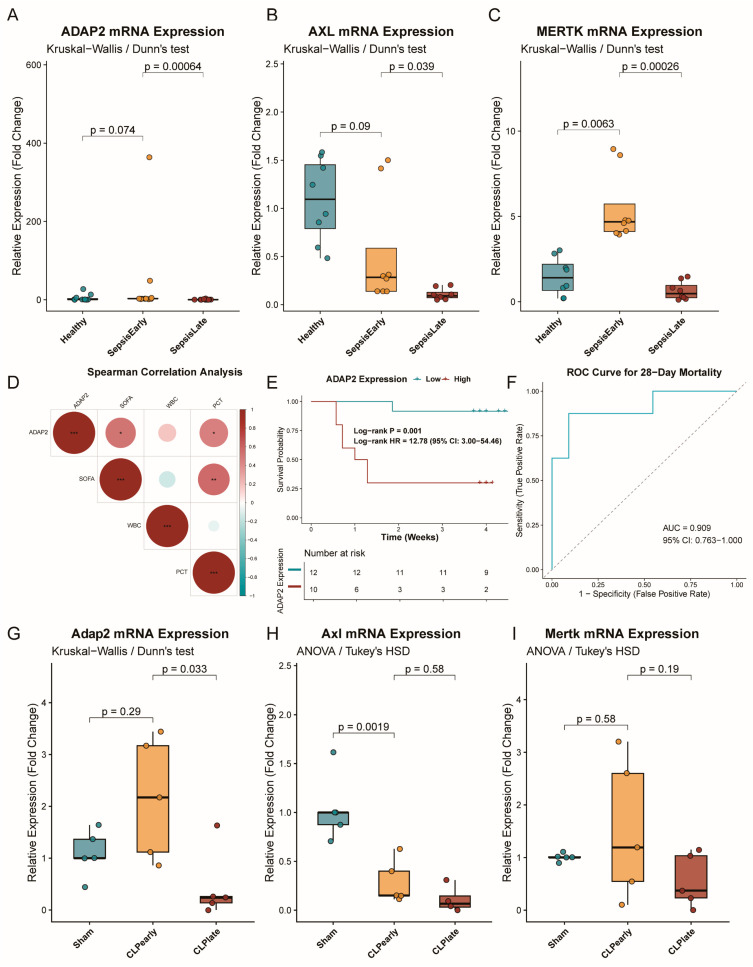
Clinical and experimental validation of ADAP2 and efferocytosis-related genes. (**A**–**C**) Relative mRNA expression of ADAP2, AXL, and MERTK in human PBMCs from Healthy, Sepsis-Early, and Sepsis-Late groups. Statistical significance was determined using the Kruskal–Wallis test followed by Dunn’s post hoc test. (**D**) Spearman correlation matrix evaluating the associations between ADAP2 expression and clinical severity indices (SOFA, WBC, PCT); * *p*  <  0.05, ** *p*  <  0.01, *** *p*  <  0.001. (**E**) Kaplan–Meier survival curves stratifying sepsis patients by high vs. low ADAP2 expression over a 4-week period. The hazard ratio (HR) and 95% confidence interval (CI) were calculated specifically using the Log-rank test. (**F**) Receiver operating characteristic (ROC) curve assessing the predictive value of ADAP2 for 28-day mortality. (**G**–**I**) Relative mRNA expression of Adap2, Axl, and Mertk in PBMCs from sham and CLP-induced septic mice (*n* = 5/group). Statistical significance was assessed via Kruskal–Wallis/Dunn’s test or ANOVA/Tukey’s HSD as indicated. Box plots display the median and interquartile range, with dots representing independent biological samples.

To evaluate clinical relevance, we quantified ADAP2, MERTK, and AXL expression in human PBMCs and CLP-induced septic mice. Consistent with our transcriptomic predictions, ADAP2 expression was maintained during early sepsis but profoundly depleted in late-stage sepsis across both human patients (Figure 7A) and the murine model (Figure 7G). MERTK and AXL exhibited similar late-stage depletion trends (Figure 7B,C,H,I). Clinically, ADAP2 expression correlated significantly with sepsis severity indices, including SOFA score, WBC count, and PCT levels (Figure 7D). Kaplan–Meier survival analysis demonstrated that patients with low ADAP2 expression experienced significantly worse 4-week survival outcomes (Log-rank *p* = 0.001; Log-rank HR = 12.78, 95% CI: 3.00–54.46) (Figure 7E). Finally, ROC analysis confirmed the robust predictive efficacy of ADAP2 for 28-day mortality (AUC = 0.909, 95% CI: 0.763–1.000) (Figure 7F). These findings validate ADAP2 as a robust prognostic candidate associated with sepsis progression.

## 4. Discussion

Sepsis is a life-threatening organ dysfunction caused by dysregulated host responses to infection, and its complex immunopathology remains a global clinical challenge [33,34,35]. The efficient clearance of apoptotic cells through efferocytosis is essential to maintain immune homeostasis and promote the resolution of inflammation [36]. In this study, we integrated multi-dimensional transcriptomic integration analyses (bulk RNA-seq, WGCNA, scRNA-seq) with machine learning algorithms to systematically identify and validate core efferocytosis-related genes in sepsis. We discovered and confirmed that ADAP2 is closely associated with macrophage-mediated efferocytosis during sepsis.

The pathology of sepsis is characterized by the extensive apoptosis of immune and tissue cells [37]. Our initial transcriptomic profiling highlighted the “Efferocytosis” pathway as significantly overrepresented. Rather than a mere byproduct of cell death, this transcriptional enrichment directly indicates an active host compensatory mechanism, aligning with previous reports that the efferocytic program is initiated early in sepsis to limit inflammatory injury [4,10,12,14].

Several previous bioinformatic studies have utilized the GSE205672 and GSE133822 cohorts to identify sepsis biomarkers, predominantly focusing on broad inflammatory pathways [38,39]. Consequently, ADAP2 and RPS17 were not previously highlighted. Through the integration of co-expression network analysis and an ensemble machine-learning framework, we successfully narrowed a complex transcriptomic landscape down to robust predictive candidates specifically linking efferocytosis to sepsis progression. The gene modules most tightly linked to efferocytosis were heavily enriched for pathways such as “phagocytosis”, “T cell-related immune regulation”, and “ribosome”. This suggests an intrinsic biological coupling between cellular material-uptake functions, adaptive immune modulation, and elevated basal metabolic activity during the acute phase of sepsis. Supporting this metabolic link, our pipeline identified RPS17, a core ribosomal protein, as a secondary consensus feature. Altered RPS17 expression may reflect underlying ribosomal stress or translational reprogramming within circulating immune subsets during sepsis. At the single-cell level, we found that pro-inflammatory (M1-like) and IFN-responsive macrophage subsets exhibited the highest efferocytic activity during early sepsis. This suggests that, at the onset of infection, efferocytosis may not be restricted to a specific polarization state but rather represents a functional property shared by multiple activated macrophage subsets to simultaneously clear apoptotic cells and limit excessive inflammation. Notably, the classic M1/M2 dichotomy does not fully capture macrophage functional heterogeneity [40]; even within M1 or IFN-γ-activated macrophages, distinct functional clusters exist. Our finding that M1-like macrophages exhibit high ADAP2 expression and elevated efferocytosis scores lends support to this emerging concept. Moreover, CellChat analysis computationally predicted active communication networks between macrophages and neutrophils or T cells, identifying putative pathways (e.g., LGALS9-CD44, ANXA1-FPR1). While we acknowledge that these inferred ligand–receptor axes are descriptive and lack direct functional validation, they provide a valuable systems-level hypothesis highlighting macrophage-mediated efferocytosis as a potential regulatory node.

Our converging evidence points to a pivotal role for ADAP2. At the bulk transcriptomic level, CIBERSORT-based immune inference indicated a strong positive correlation between ADAP2 expression and monocyte/M1 macrophage infiltration. While deconvolution algorithms may not fully capture highly aberrant sepsis phenotypes, these macroscopic trends provided a valuable baseline. Crucially, to exclude potential cell composition biases inherent in bulk transcriptomics, these observations were further investigated using high-resolution scRNA-seq analysis. This offered more definitive evidence, confirming that ADAP2 is specifically and highly expressed in monocyte/macrophage subpopulations. ADAP2 is an ArfGAP protein containing tandem PH domains; adaptor proteins of this class typically act as scaffolds in signal transduction [41], vesicular transport [42], and cytoskeletal remodeling [43]. Efferocytosis requires precise cytoskeletal reorganization to form the phagocytic cup and internalize apoptotic cells [44,45]. Accordingly, we hypothesize that ADAP2 may serves as a key linking molecule within the efferocytosis signaling cascade, potentially integrating upstream “eat-me” receptor signals (e.g., MerTK) and transmitting them to downstream cytoskeletal regulators such as Rac-family small GTPases and Cadherin-11 to drive efficient engulfment [46,47]. Finally, qPCR validation in PBMC from sepsis patients and in the CLP mouse model confirmed that ADAP2 expression changes in sepsis are consistent with our bioinformatic analyses, further reinforcing its clinical relevance and robust predictive value in the sepsis immune microenvironment. Regarding the temporal dynamics, we hypothesize that the early upregulation of ADAP2 reflects a robust acute innate immune response, potentially driven by early interferon signaling during initial infection. Conversely, the profound depletion of ADAP2 during late-stage sepsis aligns with the well-documented transition toward sepsis-induced immunosuppression. This late depletion may indicate a state of macrophage functional exhaustion, wherein the attenuation of ADAP2 and related efferocytic pathways contributes to delayed tissue repair and persistent vulnerability to secondary infections.

Clinically, quantifying ADAP2 in circulating PBMCs represents a promising minimally invasive liquid biopsy. Despite the time constraints of conventional qPCR, recent clinical validations of AI-based transcriptomic point-of-care testing (POCT) devices have demonstrated the feasibility of rapid, bedside host-response profiling in critical care workflows [48]. Its profound downregulation may serve as a surrogate transcriptomic indicator of impaired efferocytosis and the transition toward sepsis-induced immunosuppression [49]. Furthermore, our expanded clinical validation revealed that ADAP2 expression correlates significantly with clinical severity indices (e.g., SOFA score), and exhibits robust predictive efficacy for 28-day mortality. Unlike these general inflammatory markers, ADAP2 uniquely reflects macrophage functional states. Integrating ADAP2 into multi-marker POCT panels could substantially enhance prognostic stratification and guide early personalized immunomodulation.

This study has several limitations. First, the analysis was based entirely on publicly available bioinformatic datasets, so the findings are inherently correlative. The direct causal link between ADAP2 and efferocytosis must be validated by subsequent in vitro and in vivo functional experiments. Second, the absence of protein-level validation and longitudinal sampling limits the translational interpretation of our findings. Third, sepsis is highly heterogeneous, and our analyses rely on specific cohorts; therefore, the generalizability of the results to patients with different pathogens or infection sites remains to be confirmed. In addition, our work is restricted to peripheral blood molecular signatures; extending the investigation of ADAP2 to tissue-resident macrophages, particularly alveolar macrophages in sepsis-induced acute lung injury, is essential to determine whether the peripheral blood signatures identified in this study translate to organ-level immunopathology.

## 5. Conclusions

In conclusion, to address the limitations of conventional biomarkers in reflecting specific immune functional states, this study integrated multi-omics and machine learning to identify efferocytosis-related prognostic features. We identified ADAP2 as a highly conserved candidate. Its specific depletion in macrophages during sepsis strongly correlates with reduced efferocytosis signatures and heightened clinical severity across human and murine cohorts. While future functional validation remains necessary to establish causality, these findings highlight ADAP2 as a robust transcriptomic candidate biomarker for early sepsis stratification.

## Data Availability

The datasets used and analyzed during the current study are available from the corresponding author on reasonable request. The custom R scripts used for the bioinformatic analyses in this study are provided as Supplementary Material.

## Supplementary Materials

The following supporting information can be downloaded at https://www.mdpi.com/article/10.3390/pathogens15060596/s1, Figure S1: Soft threshold selection and co-expression module identification for WGCNA; Figure S2: Detailed feature selection metrics across machine learning algorithms; Figure S3: Single-cell data integration, annotation validation, and functional landscape of ADAP2+ macrophages in sepsis; Figure S4: Trajectory analysis reveals the dynamic exhaustion of ADAP2+ macrophages in late-stage sepsis; Figure S5: CellChat analysis of immune cells in sepsis; Table S1: DataSet; Table S2: Gene list; Table S3: Cross-validated performance of the five machine learning algorithms; Table S4: ML Interpretability; Table S5: QPCR primer sequence.

## Abbreviations

The following abbreviations are used in this manuscript:

ADAP2	ArfGAP with dual PH domains 2
ANOVA	Analysis of variance
ArfGAP	ADP-ribosylation factor GTPase-activating protein
AUC	Area under the receiver operating characteristic curve
BP	Biological process
CC	Cellular component
CIBERSORT	Cell-type identification by estimating relative subsets of RNA transcripts
CLP	Cecum ligation and puncture
DAMP	Damage-associated molecular pattern
DEG	Differentially expressed gene
FDR	False discovery rate
GEO	Gene Expression Omnibus
GO	Gene Ontology
GS	Gene significance
HMGB1	High mobility group box 1
ISG	Interferon-stimulated gene
KEGG	Kyoto Encyclopedia of Genes and Genomes
MAD	Median absolute deviation
MF	Molecular function
MM	Module membership
PBMC	Peripheral blood mononuclear cell
PCA	Principal component analysis
qRT-PCR	Quantitative reverse transcription polymerase chain reaction
RNA-seq	RNA sequencing
ROC	Receiver operating characteristic
scRNA-seq	Single-cell RNA sequencing
ssGSEA	Single-sample gene set enrichment analysis
TOM	Topological overlap matrix
WGCNA	Weighted gene co-expression network analysis
