# Supplementary material for: Incorporating WGCNA and Machine Learning to Identify ADAP2 as a Critical Efferocytosis-Related Gene in Sepsis
Source: Pathogens. 2026 Jun 1;15(6):596. doi: 10.3390/pathogens15060596 (PMC13304750; doi:10.3390/pathogens15060596)
Supplement: Supplementary file 1 [file pathogens-15-00596-s001.zip › Supplementary Figures.pdf]

Figure S1

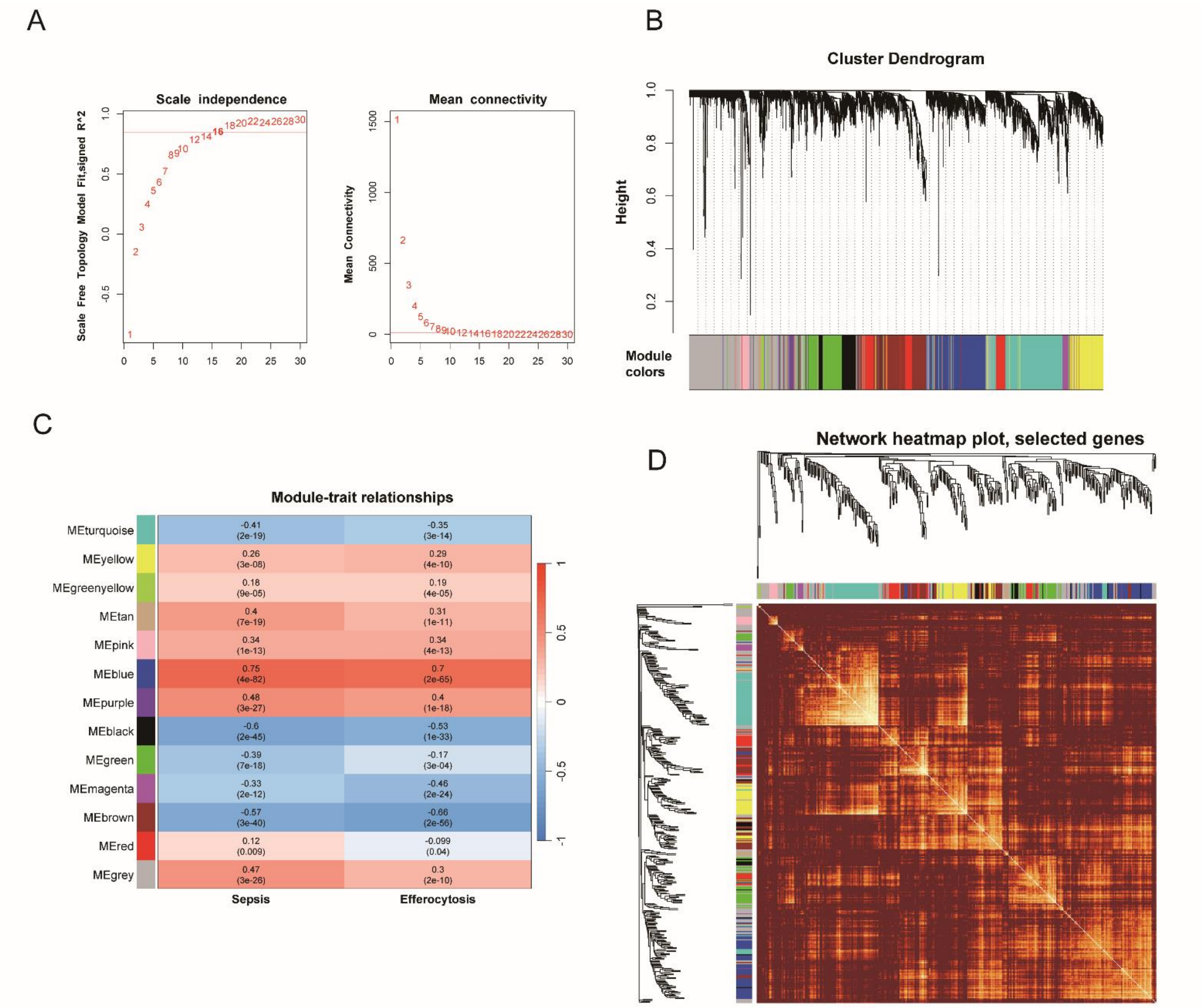

Figure S1 Soft threshold selection and co-expression module identification for WGCNA. **A** Plots for determining the optimal soft threshold: Left, Scale independence; Right, Mean connectivity. The soft threshold $\beta = 16$ was selected, as it balances the fit of scale-free topology and network connectivity. **B** The Dynamic Tree Cut algorithm partitioned genes into 13 co-expression modules, with corresponding module colors annotated below the dendrogram. **C** Module-trait correlation heatmap; 13 co-expression modules (Dynamic Tree Cut) are shown (color intensity = correlation coefficient). The blue module strongly correlates with Sepsis ($r = 0.72$, $p = 4e-82$) and Efferocytosis ($r = 0.7$, $p = 2e-6$); the black/brown modules also show significant associations. **D** Topological Overlap Matrix (TOM) network heatmap (bottom) and hierarchical gene clustering tree (top) from WGCNA.

Figure S2

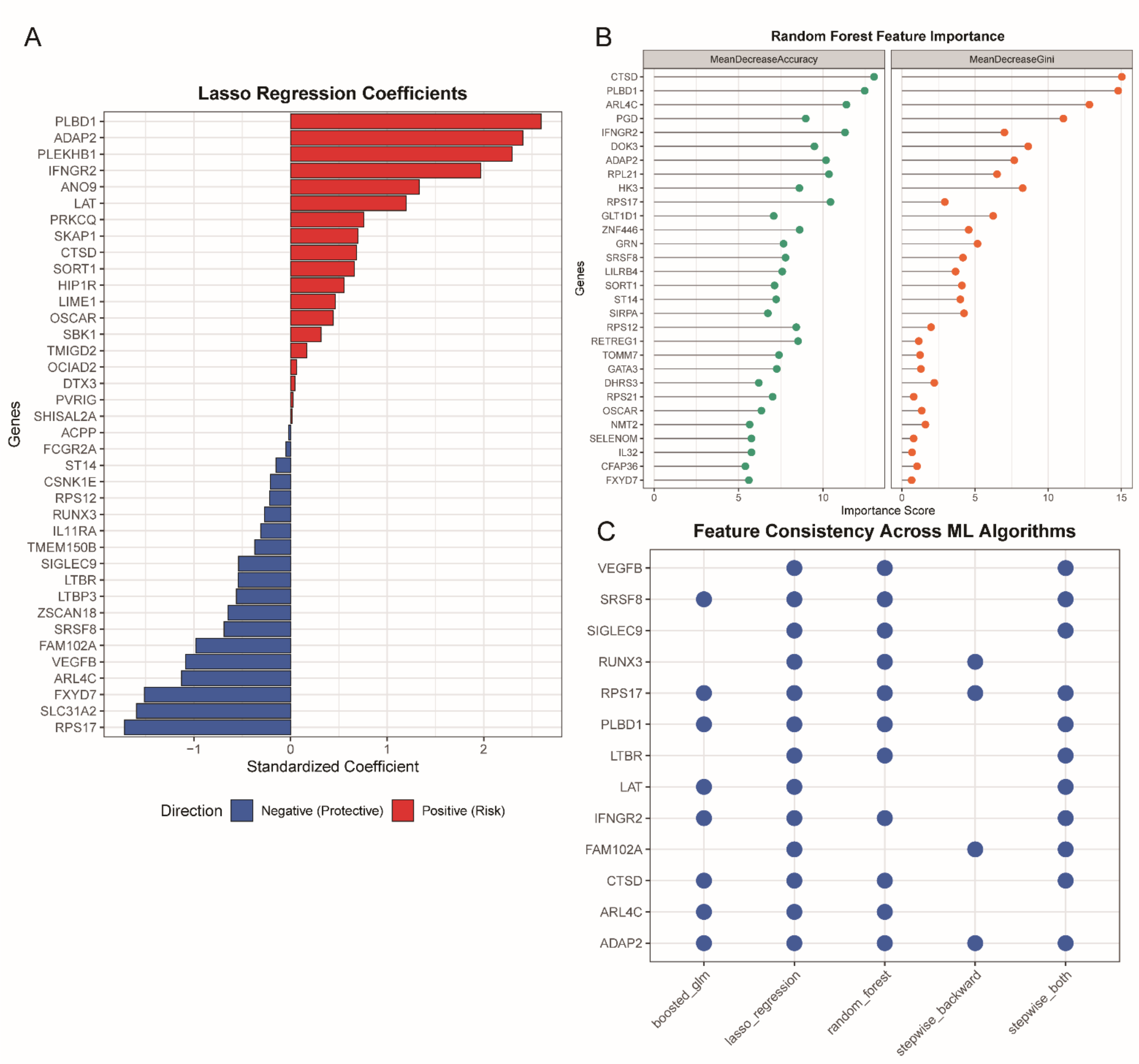

Figure S2. Detailed feature selection metrics across machine learning algorithms. (**A**) Standardized coefficients of features selected by Lasso regression. Positive values (red) indicate risk factors associated with sepsis, while negative values (blue) indicate protective factors. (**B**) Feature importance ranking derived from the Random Forest model, evaluated by Mean Decrease Accuracy and Mean Decrease Gini. (**C**) Consensus matrix illustrating the selection consistency of candidate genes across five distinct machine learning algorithms (GlmBoost, Lasso, Random Forest, Stepwise backward, and Stepwise both).

Figure S3

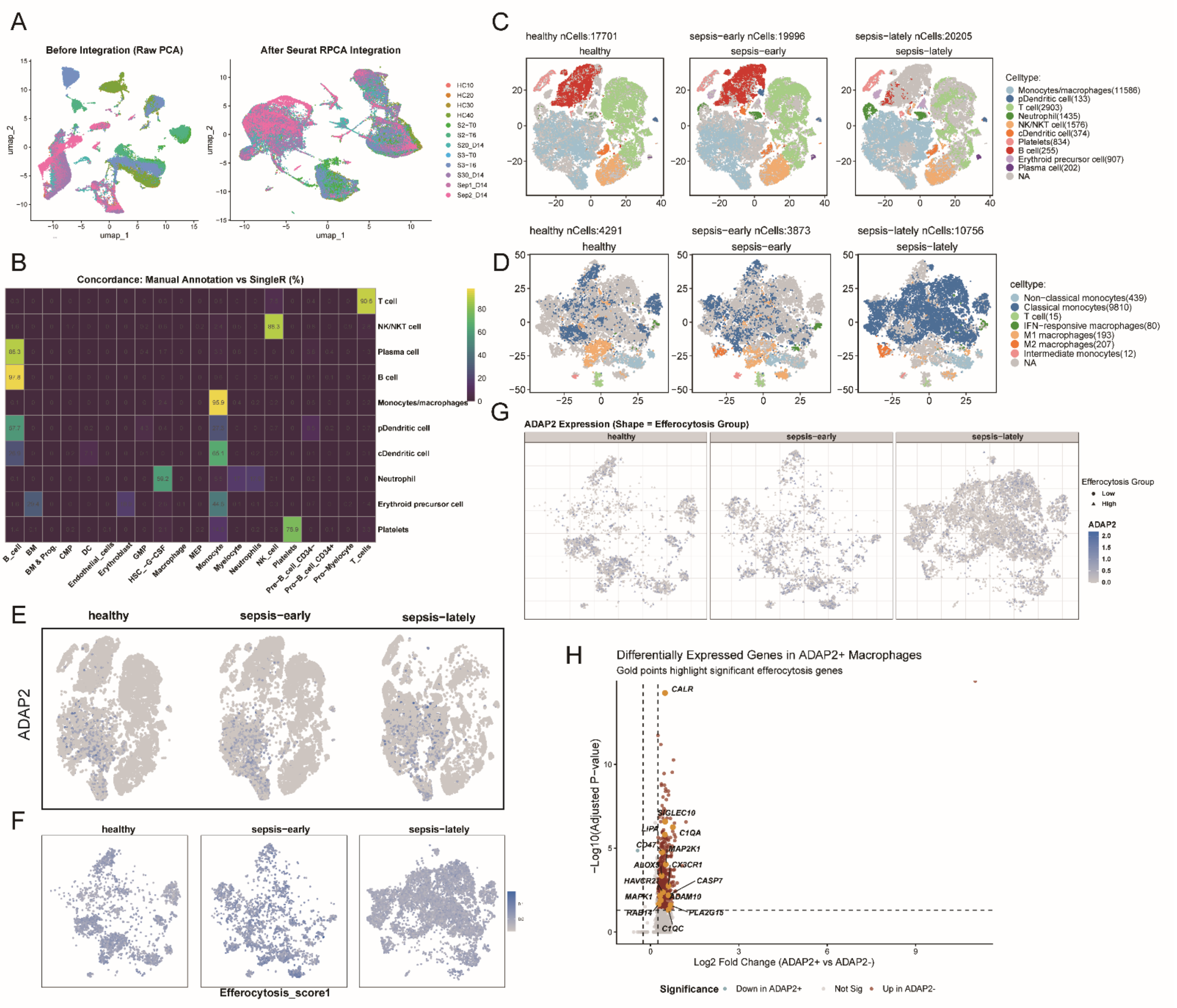

Figure S3 Single-cell data integration, annotation validation, and functional landscape of ADAP2+ macrophages in sepsis. (A) UMAP visualizations comparing data structure before (Raw PCA, left) and after Seurat reciprocal PCA (RPCA) integration (right), demonstrating the effective mitigation of batch effects across different sequencing samples. (B) Confusion matrix heatmap illustrating the high concordance (%) between our manual canonical marker-based cell annotations and the automated predictions generated by SingleR. (C) Group-stratified UMAP plots of global peripheral blood mononuclear cell (PBMC) clusters across healthy, sepsis-early, and sepsis-lately clinical stages. (D) UMAP plots of the monocyte/macrophage subclustering stratified by clinical stage, identifying distinct subsets including non-classical, classical, and intermediate monocytes, alongside IFN-responsive, M1, and M2 macrophages. (E) UMAP visualization of ADAP2 expression within the monocyte/macrophage lineage across the three clinical stages. Darker blue indicates higher expression levels. (F) UMAP plots depicting the efferocytosis-related gene module scores (calculated based on Table S1) across the three clinical stages. Darker blue indicates higher functional enrichment scores. (G) Co-localization UMAP plots visualizing both ADAP2 expression levels (color gradient) and discrete efferocytosis functional groups (Shape: Square = Low, Triangle = High) across the healthy, sepsis-early, and sepsis-lately stages. (H) Volcano plot displaying the differentially expressed genes between ADAP2+ and ADAP2- macrophages. Gold points highlight the significantly upregulated core genes associated with the efferocytosis pathway.

Figure S4

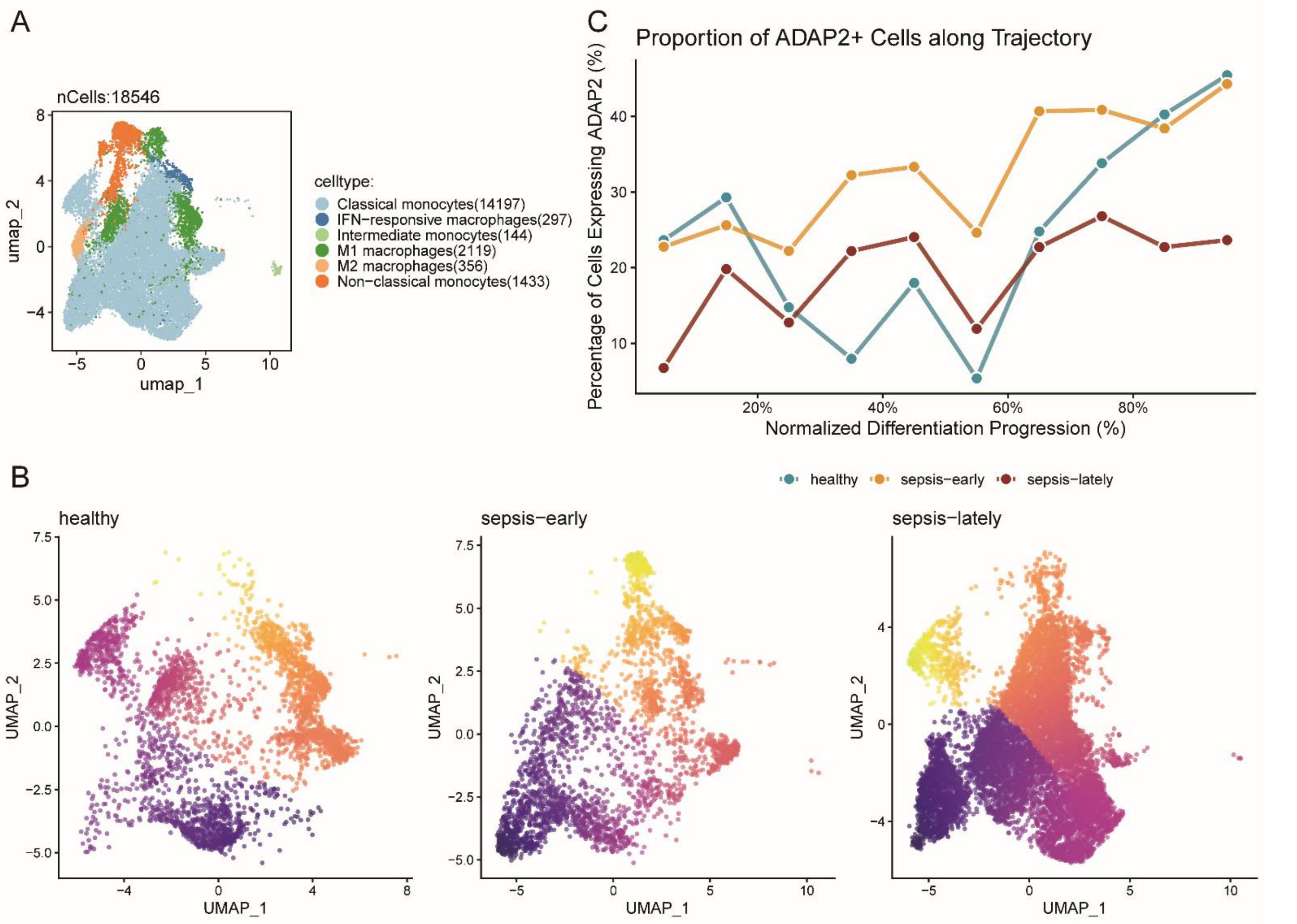

Figure S4. Trajectory analysis reveals the dynamic exhaustion of ADAP2+ macrophages in late-stage sepsis. (A) UMAP embedding of the isolated pro-inflammatory and IFN-responsive macrophage lineage. (B) Independent Slingshot pseudotime trajectories for healthy, early-stage, and late-stage sepsis, anchored at classical monocytes. (C) Dynamic proportion of ADAP2+ cells along the normalized differentiation axis (0%–100%, 10% bins). Early-stage sepsis shows robust ADAP2 induction, whereas late-stage sepsis exhibits a blunted response and terminal depletion.

Figure S5

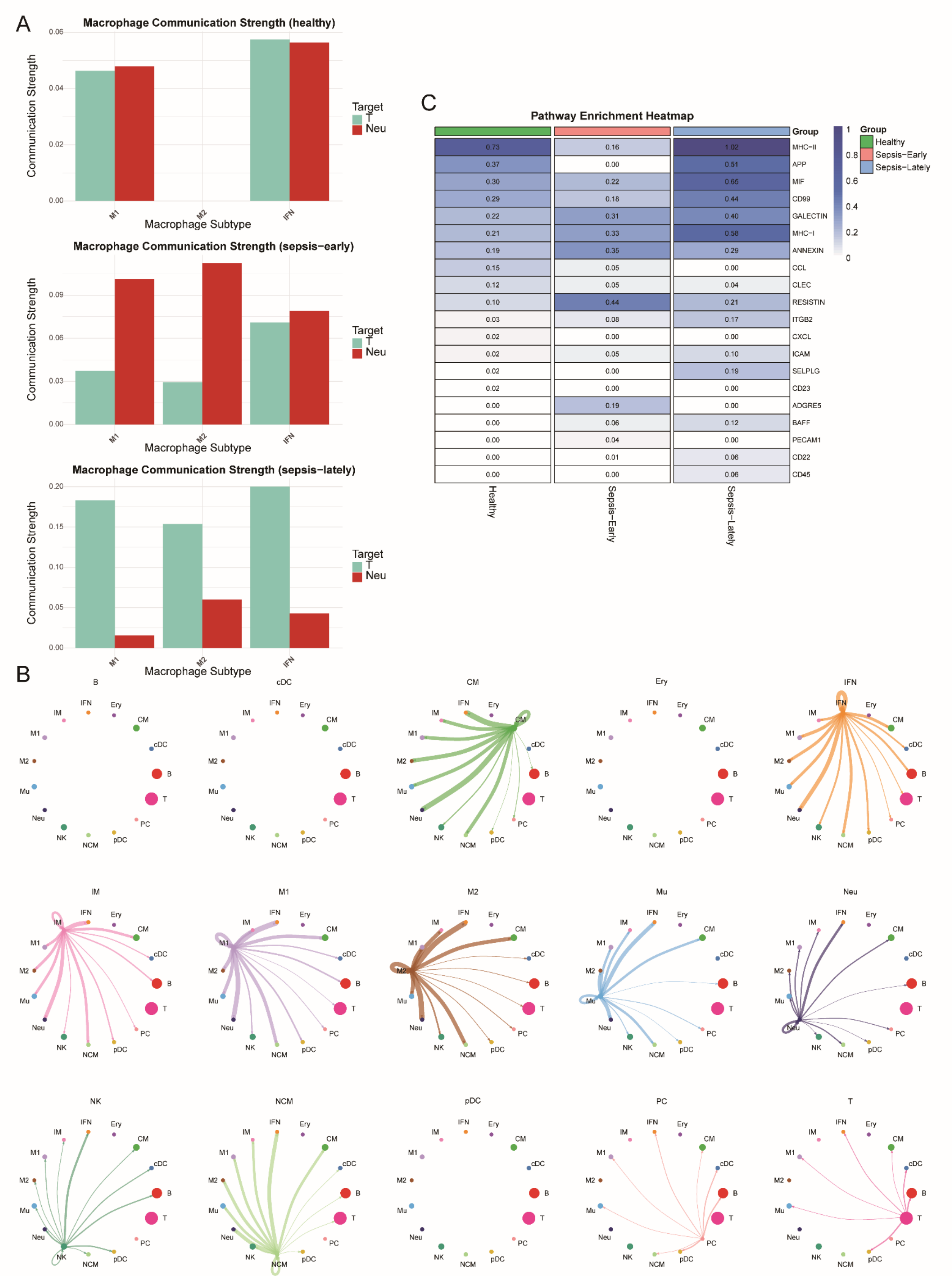

Figure S5 CellChat analysis of immune cells in sepsis. **A** Bar plots of macrophage-related communication strength with other cell types, stratified by Healthy, Sepsis-Early, and Sepsis-Late groups. **B** Chord diagrams of directional intercellular communication for Sepsis-Early, displaying signal flow between cell types (distinct colors

correspond to cell subsets). **C** Pathway enrichment heatmap; rows represent enriched pathways, columns represent groups, and color intensity indicates pathway enrichment level.
