# Supplementary material for: Incorporating WGCNA and Machine Learning to Identify ADAP2 as a Critical Efferocytosis-Related Gene in Sepsis
Source: Pathogens. 2026 Jun 1;15(6):596. doi: 10.3390/pathogens15060596 (PMC13304750; doi:10.3390/pathogens15060596)
Supplement: Supplementary file 1 [file pathogens-15-00596-s001.zip › Supplementary Tables.pdf]

Supplementary Table S1 DataSet

GEO_ID	type	Platform	Organism	Source	Criteria used to define sepsis	Age	Samples	Timing of biosample collection
GSE205672	RNA-seq	Illumina NovaSeq 6000	Homo sapiens	PBMC/ monocyte	ACCP/ SCCM	59.3 ±20.7	161 sepsis and 299 healthy controls	Same day
GSE133822	RNA-seq	Illumina HiSeq 2500	Homo sapiens	Monocyte	ACCP/ SCCM	Mean 60	30 sepsis and 20 controls	after at least 24–48 h of sepsis onset and typically between 2 and 9 d after sepsis onset.
GSE175453	scRNA-seq	Illumina NextSeq 500/NovaSeq 6000	Homo sapiens	PBMC	Sepsis-3	Mean 65	4 late sepsis and 2 controls	post-sepsis day 14-21
GSE167363	scRNA-seq	Illumina NovaSeq 6000	Homo sapiens	PBMC	Sepsis-3	Range 45-75	4 early septic and 2 controls	Within 6 hours after sepsis identification

Supplementary Table S2 gene list

Efferocytes-Related Genes

Gene ID	Gene Symbol	Description
100133941	CD24	CD24 molecule [KO:K06469]
10062	NR1H3	nuclear receptor subfamily 1 group H member 3 [KO:K08536]
102	ADAM10	ADAM metallopeptidase domain 10 [KO:K06704] [EC:3.4.24.81]
10326	SIRPB1	signal regulatory protein beta 1 [KO:K06551]
10396	ATP8A1	ATPase phospholipid transporting 8A1 [KO:K14802] [EC:7.6.2.1]
10461	MERTK	MER proto-oncogene, tyrosine kinase [KO:K05117] [EC:2.7.10.1]
1051	CEBPB	CCAAT enhancer binding protein beta [KO:K10048]
114786	XKR4	XK related 4 [KO:K26811]
121601	ANO4	anoctamin 4 [KO:K19499]
126129	CPT1C	carnitine palmitoyltransferase 1C [KO:K19524] [EC:2.3.1.21]
1374	CPT1A	carnitine palmitoyltransferase 1A [KO:K08765] [EC:2.3.1.21]
1375	CPT1B	carnitine palmitoyltransferase 1B [KO:K19523] [EC:2.3.1.21]
1385	CREB1	cAMP responsive element binding protein 1 [KO:K05870]
1398	CRK	CRK proto-oncogene, adaptor protein [KO:K04438]
1399	CRKL	CRK like proto-oncogene, adaptor protein [KO:K04438]
140885	SIRPA	signal regulatory protein alpha [KO:K06551]
1432	MAPK14	mitogen-activated protein kinase 14 [KO:K04441] [EC:2.7.11.24]
1524	CX3CR1	C-X3-C motif chemokine receptor 1 [KO:K04192]
161291	TMEM30B	transmembrane protein 30B [KO:K26935]
177	AGER	advanced glycosylation end-product specific receptor [KO:K19722]
1788	DNMT3A	DNA methyltransferase 3 alpha [KO:K17398] [EC:2.1.1.37]
1793	DOCK1	dedicator of cytokinesis 1 [KO:K13708]

1844	DUSP2	dual specificity phosphatase 2 [KO:K04459] [EC:3.1.3.16 3.1.3.48]
1846	DUSP4	dual specificity phosphatase 4 [KO:K04459] [EC:3.1.3.16 3.1.3.48]
1847	DUSP5	dual specificity phosphatase 5 [KO:K04459] [EC:3.1.3.16 3.1.3.48]
1849	DUSP7	dual specificity phosphatase 7 [KO:K04459] [EC:3.1.3.16 3.1.3.48]
1850	DUSP8	dual specificity phosphatase 8 [KO:K04459] [EC:3.1.3.16 3.1.3.48]
19	ABCA1	ATP binding cassette subfamily A member 1 [KO:K05641]
1901	S1PR1	sphingosine-1-phosphate receptor 1 [KO:K04288]
196527	ANO6	anoctamin 6 [KO:K19500]
203859	ANO5	anoctamin 5 [KO:K19480]
2056	EPO	erythropoietin [KO:K05437]
2057	EPOR	erythropoietin receptor [KO:K05079]
23166	STAB1	stabilin 1 [KO:K19020]
23200	ATP11B	ATPase phospholipid transporting 11B (putative) [KO:K26934]
23250	ATP11A	ATPase phospholipid transporting 11A [KO:K26934]
23339	VPS39	VPS39 subunit of HOPS complex [KO:K20183]
23355	VPS8	VPS8 subunit of CORVET complex [KO:K20178]
23411	SIRT1	sirtuin 1 [KO:K11411] [EC:2.3.1.286]
23659	PLA2G15	phospholipase A2 group XV [KO:K06129] [EC:3.1.1.5]
240	ALOX5	arachidonate 5-lipoxygenase [KO:K00461] [EC:1.13.11.34]
24145	PANX1	pannexin 1 [KO:K03443]
246	ALOX15	arachidonate 15-lipoxygenase [KO:K00460] [EC:1.13.11.33]
2621	GAS6	growth arrest specific 6 [KO:K05464]
26762	HAVCR1	hepatitis A virus cellular receptor 1 [KO:K20413]
27072	VPS41	VPS41 subunit of HOPS complex [KO:K20184]
286046	XKR6	XK related 6 [KO:K26811]
286410	ATP11C	ATPase phospholipid transporting 11C [KO:K26934]
29933	GPR132	G protein-coupled receptor 132 [KO:K08426]
3091	HIF1A	hypoxia inducible factor 1 subunit alpha [KO:K08268]
338382	RAB7B	RAB7B, member RAS oncogene family [KO:K07898]
338440	ANO9	anoctamin 9 [KO:K19503]
343702	XKR7	XK related 7 [KO:K26811]
3586	IL10	interleukin 10 [KO:K05443]
3685	ITGAV	integrin subunit alpha V [KO:K06487]
3690	ITGB3	integrin subunit beta 3 [KO:K06493]
3693	ITGB5	integrin subunit beta 5 [KO:K06588]
3717	JAK2	Janus kinase 2 [KO:K04447] [EC:2.7.10.2]
383	ARG1	arginase 1 [KO:K01476] [EC:3.5.3.1]
384	ARG2	arginase 2 [KO:K01476] [EC:3.5.3.1]
389668	XKR9	XK related 9 [KO:K26814]
3988	LIPA	lipase A, lysosomal acid type [KO:K01052] [EC:3.1.1.13]
4035	LRP1	LDL receptor related protein 1 [KO:K04550]
405	ARNT	aryl hydrocarbon receptor nuclear translocator [KO:K09097]
4240	MFGE8	milk fat globule EGF and factor V/VIII domain containing [KO:K17253]

4772	NFATC1	nuclear factor of activated T cells 1 [KO:K04446]
4773	NFATC2	nuclear factor of activated T cells 2 [KO:K17332]
4775	NFATC3	nuclear factor of activated T cells 3 [KO:K17333]
4776	NFATC4	nuclear factor of activated T cells 4 [KO:K17334]
487	ATP2A1	ATPase sarcoplasmic/endoplasmic reticulum Ca2+ transporting 1 [KO:K05853] [EC:7.2.2.10]
488	ATP2A2	ATPase sarcoplasmic/endoplasmic reticulum Ca2+ transporting 2 [KO:K05853] [EC:7.2.2.10]
489	ATP2A3	ATPase sarcoplasmic/endoplasmic reticulum Ca2+ transporting 3 [KO:K05853] [EC:7.2.2.10]
4953	ODC1	ornithine decarboxylase 1 [KO:K01581] [EC:4.1.1.17]
5029	P2RY2	purinergic receptor P2Y2 [KO:K04269]
5031	P2RY6	pyrimidinergic receptor P2Y6 [KO:K04272]
50636	ANO7	anoctamin 7 [KO:K19501]
5087	PBX1	PBX homeobox 1 [KO:K09355]
51454	GULP1	GULP PTB domain containing engulfment adaptor 1 [KO:K23285]
51552	RAB14	RAB14, member RAS oncogene family [KO:K07881]
5175	PECAM1	platelet and endothelial cell adhesion molecule 1 [KO:K06471]
51761	ATP8A2	ATPase phospholipid transporting 8A2 [KO:K14802] [EC:7.6.2.1]
5467	PPARD	peroxisome proliferator activated receptor delta [KO:K04504]
5468	PPARG	peroxisome proliferator activated receptor gamma [KO:K08530]
54896	SLC66A1	solute carrier family 66 member 1 [KO:K23678]
55113	XKR8	XK related 8 [KO:K26813]
55423	SIRPG	signal regulatory protein gamma [KO:K06551]
55576	STAB2	stabilin 2 [KO:K19013]
55754	TMEM30A	transmembrane protein 30A [KO:K26935]
558	AXL	AXL receptor tyrosine kinase [KO:K05115] [EC:2.7.10.1]
55823	VPS11	VPS11 core subunit of CORVET and HOPS complexes [KO:K20179]
5594	MAPK1	mitogen-activated protein kinase 1 [KO:K04371] [EC:2.7.11.24]
5595	MAPK3	mitogen-activated protein kinase 3 [KO:K04371] [EC:2.7.11.24]
5600	MAPK11	mitogen-activated protein kinase 11 [KO:K04441] [EC:2.7.11.24]
5603	MAPK13	mitogen-activated protein kinase 13 [KO:K04441] [EC:2.7.11.24]
5604	MAP2K1	mitogen-activated protein kinase kinase 1 [KO:K04368] [EC:2.7.12.2]
5605	MAP2K2	mitogen-activated protein kinase kinase 2 [KO:K04369] [EC:2.7.12.2]
5627	PROS1	protein S [KO:K03908]
56848	SPHK2	sphingosine kinase 2 [KO:K04718] [EC:2.7.1.91]
5732	PTGER2	prostaglandin E receptor 2 [KO:K04259]
5734	PTGER4	prostaglandin E receptor 4 [KO:K04261]
5743	PTGS2	prostaglandin-endoperoxide synthase 2 [KO:K11987] [EC:1.14.99.1]
5747	PTK2	protein tyrosine kinase 2 [KO:K05725] [EC:2.7.10.2]
575	ADGRB1	adhesion G protein-coupled receptor B1 [KO:K04596]
57617	VPS18	VPS18 core subunit of CORVET and HOPS complexes [KO:K20181]

5777	PTPN6	protein tyrosine phosphatase non-receptor type 6 [KO:K05697] [EC:3.1.3.48]
5781	PTPN11	protein tyrosine phosphatase non-receptor type 11 [KO:K07293] [EC:3.1.3.48]
5868	RAB5A	RAB5A, member RAS oncogene family [KO:K07887]
5869	RAB5B	RAB5B, member RAS oncogene family [KO:K07888]
5878	RAB5C	RAB5C, member RAS oncogene family [KO:K07889]
5879	RAC1	Rac family small GTPase 1 [KO:K04392]
6256	RXRA	retinoid X receptor alpha [KO:K08524]
6300	MAPK12	mitogen-activated protein kinase 12 [KO:K04441] [EC:2.7.11.24]
6376	CX3CL1	C-X3-C motif chemokine ligand 1 [KO:K05508]
63982	ANO3	anoctamin 3 [KO:K19498]
64284	RAB17	RAB17, member RAS oncogene family [KO:K07909]
6446	SGK1	serum/glucocorticoid regulated kinase 1 [KO:K13302] [EC:2.7.11.1]
64601	VPS16	VPS16 core subunit of CORVET and HOPS complexes [KO:K20180]
64805	P2RY12	purinergic receptor P2Y12 [KO:K04298]
65082	VPS33A	VPS33A core subunit of CORVET and HOPS complexes [KO:K20182]
6513	SLC2A1	solute carrier family 2 member 1 [KO:K07299]
6566	SLC16A1	solute carrier family 16 member 1 [KO:K08179]
682	BSG	basigin (Ok blood group) [KO:K06535]
6868	ADAM17	ADAM metallopeptidase domain 17 [KO:K06059] [EC:3.4.24.86]
7040	TGFB1	transforming growth factor beta 1 [KO:K13375]
7057	THBS1	thrombospondin 1 [KO:K16857]
712	C1QA	complement C1q A chain [KO:K03986]
713	C1QB	complement C1q B chain [KO:K03987]
714	C1QC	complement C1q C chain [KO:K03988]
7301	TYRO3	TYRO3 protein tyrosine kinase [KO:K05116] [EC:2.7.10.1]
7376	NR1H2	nuclear receptor subfamily 1 group H member 2 [KO:K08535]
7386	UQCRFS1	ubiquinol-cytochrome c reductase, Rieske iron-sulfur polypeptide 1 [KO:K00411] [EC:7.1.1.8]
7879	RAB7A	RAB7A, member RAS oncogene family [KO:K07897]
80824	DUSP16	dual specificity phosphatase 16 [KO:K04459] [EC:3.1.3.16 3.1.3.48]
811	CALR	calreticulin [KO:K08057]
815	CAMK2A	calcium/calmodulin dependent protein kinase II alpha [KO:K04515] [EC:2.7.11.17]
816	CAMK2B	calcium/calmodulin dependent protein kinase II beta [KO:K04515] [EC:2.7.11.17]
817	CAMK2D	calcium/calmodulin dependent protein kinase II delta [KO:K04515] [EC:2.7.11.17]
818	CAMK2G	calcium/calmodulin dependent protein kinase II gamma [KO:K04515] [EC:2.7.11.17]
834	CASP1	caspase 1 [KO:K01370] [EC:3.4.22.36]
836	CASP3	caspase 3 [KO:K02187] [EC:3.4.22.56]
8398	PLA2G6	phospholipase A2 group VI [KO:K16343] [EC:3.1.1.4]
840	CASP7	caspase 7 [KO:K04397] [EC:3.4.22.60]

84465	MEGF11	multiple EGF like domains 11 [KO:K24068]
84466	MEGF10	multiple EGF like domains 10 [KO:K24068]
84868	HAVCR2	hepatitis A virus cellular receptor 2 [KO:K20414]
8578	SCARF1	scavenger receptor class F member 1 [KO:K24318]
8754	ADAM9	ADAM metallopeptidase domain 9 [KO:K06834] [EC:3.4.24.-]
8877	SPHK1	sphingosine kinase 1 [KO:K04718] [EC:2.7.1.91]
89790	SIGLEC10	sialic acid binding Ig like lectin 10 [KO:K06749]
9023	CH25H	cholesterol 25-hydroxylase [KO:K10223] [EC:1.14.99.38]
91937	TIMD4	T cell immunoglobulin and mucin domain containing 4 [KO:K26936]
9261	MAPKAPK2	MAPK activated protein kinase 2 [KO:K04443] [EC:2.7.11.1]
9392	TGFBRAP1	transforming growth factor beta receptor associated protein 1 [KO:K20177]
948	CD36	CD36 molecule (CD36 blood group) [KO:K06259]
9564	BCAR1	BCAR1 scaffold protein, Cas family member [KO:K05726]
961	CD47	CD47 molecule [KO:K06266]
9844	ELMO1	engulfment and cell motility 1 [KO:K12366]

Supplementary Table S3 Cross-validated performance of the five machine learning algorithms

Model	CV-AUC (95% CI)	Accuracy	Sensitivity	Specificity
LASSO	0.991 (0.984-0.998)	0.971	0.983	0.933
Boosted_GLM	0.975 (0.961-0.989)	0.932	0.97	0.847
Random_Forest	0.972 (0.954-0.989)	0.932	0.966	0.86
Stepwise_Both	0.943 (0.918-0.967)	0.941	0.973	0.9

Notes: Abbreviations: CV-AUC, cross-validated area under the receiver operating characteristic curve; CI, confidence interval; LASSO, least absolute shrinkage and selection operator; GLM, generalized linear model. All performance metrics (including CV-AUC, accuracy, sensitivity, and specificity) represent the average out-of-fold results calculated across the 10 resamples. This internal validation framework was utilized to provide an unbiased evaluation of the models' robust ability to distinguish patients with sepsis from healthy controls. Models are ranked in descending order of their CV-AUC values.

Supplementary Table S4 ML Interpretability

Gene	Coefficient	Mean Decrease Accuracy	Mean Decrease Gini	lasso_regression	boosted_glm	random_forest	stepwise_both	stepwise_backward	Confidence_Tier
RPS17	-1.71818	10.44277	2.930848	1	1	1	1	1	Strict Consensus (5/5)
ADAP2	2.404469	10.17845	7.67311	1	1	1	1	1	Strict Consensus (5/5)
CTSD	0.684968	13.0179	15.03128	1	1	1	1	0	High Confidence (3-4/5)
PLBD1	2.592892	12.46535	14.77954	1	1	1	1	0	High Confidence (3-4/5)

IFNGR2	1.968483	11.30371	7.005277	1	1	1	1	0	High Confidence (3-4/5)
SRSF8	-0.68884	7.778812	4.16938	1	1	1	1	0	High Confidence (3-4/5)
ARL4C	-1.12993	11.38389	12.80888	1	1	1	0	0	High Confidence (3-4/5)
RUNX3	-0.26984	5.010394	0.657015	1	0	1	0	1	High Confidence (3-4/5)
VEGFB	-1.08644	4.97589	0.57189	1	0	1	1	0	High Confidence (3-4/5)
LTBR	-0.54304	3.803007	1.291582	1	0	1	1	0	High Confidence (3-4/5)
LAT	1.196928	3.07258	0.899036	1	1	0	1	0	High Confidence (3-4/5)
SIGLEC9	-0.53856	2.879185	1.688736	1	0	1	1	0	High Confidence (3-4/5)
FAM102A	-0.97877	2.790952	0.282994	1	0	0	1	1	High Confidence (3-4/5)
PGD	0	8.976322	11.03398	0	0	1	0	1	Algorithm-Specific (1-2/5)
RPS12	-0.21808	8.414876	1.989824	1	0	1	0	0	Algorithm-Specific (1-2/5)
GATA3	0	7.260702	1.299032	1	0	1	0	0	Algorithm-Specific (1-2/5)
ST14	-0.15096	7.22459	3.991516	1	0	1	0	0	Algorithm-Specific (1-2/5)
SORT1	0.660314	7.129708	4.098163	1	0	1	0	0	Algorithm-Specific (1-2/5)
GLT1D1	0	7.080367	6.233464	0	0	1	0	1	Algorithm-Specific (1-2/5)
OSCAR	0.44278	6.352189	1.354367	1	0	1	0	0	Algorithm-Specific (1-2/5)

PTPRCAP	0	3.774428	0.575187	0	1	1	0	0	Algorithm-Specific (1-2/5)
SLC31A2	-1.59444	3.600721	0.326197	1	0	0	1	0	Algorithm-Specific (1-2/5)
PRKCQ	0.759052	3.566517	0.472787	1	0	0	0	1	Algorithm-Specific (1-2/5)
HIP1R	0.554237	3.383818	0.415581	1	0	0	0	1	Algorithm-Specific (1-2/5)
ZSCAN18	-0.64604	3.17391	0.577194	1	0	1	0	0	Algorithm-Specific (1-2/5)
PLEKHB1	2.295086	2.692728	1.17814	1	0	0	0	1	Algorithm-Specific (1-2/5)
GZMM	0	2.60357	0.260512	1	0	0	0	1	Algorithm-Specific (1-2/5)
DTX3	0.046205	2.564846	0.468666	1	0	0	0	1	Algorithm-Specific (1-2/5)
TMEM150B	-0.37163	2.103316	0.279311	1	0	0	0	1	Algorithm-Specific (1-2/5)
RPL21	0	10.35215	6.500506	0	0	1	0	0	Algorithm-Specific (1-2/5)
DOK3	0	9.488441	8.625405	0	0	1	0	0	Algorithm-Specific (1-2/5)
ZNF446	0	8.615234	4.562019	0	0	1	0	0	Algorithm-Specific (1-2/5)
HK3	0	8.599686	8.252563	0	0	1	0	0	Algorithm-Specific (1-2/5)
RETREG1	0	8.520712	1.149089	0	0	1	0	0	Algorithm-Specific (1-2/5)
GRN	0	7.662129	5.166128	0	0	1	0	0	Algorithm-Specific (1-2/5)
LILRB4	0	7.586849	3.664049	0	0	1	0	0	Algorithm-Specific (1-2/5)

TOMM7	0	7.391093	1.237119	0	0	1	0	0	Algorithm-Specific (1-2/5)
RPS21	0	7.016899	0.804809	0	0	1	0	0	Algorithm-Specific (1-2/5)
SIRPA	0	6.734393	4.243354	0	0	1	0	0	Algorithm-Specific (1-2/5)
DHRS3	0	6.198414	2.211046	0	0	1	0	0	Algorithm-Specific (1-2/5)
NMT2	0	5.654692	1.607735	0	0	1	0	0	Algorithm-Specific (1-2/5)
FXYD7	-1.5131	5.60891	0.663519	1	0	0	0	0	Algorithm-Specific (1-2/5)
CFAP36	0	5.400832	1.031601	0	0	1	0	0	Algorithm-Specific (1-2/5)
APBA2	0	5.38787	1.676608	0	0	1	0	0	Algorithm-Specific (1-2/5)
PTAFR	0	5.348612	2.479123	0	0	1	0	0	Algorithm-Specific (1-2/5)
RPS27A	0	5.340522	0.917431	0	0	1	0	0	Algorithm-Specific (1-2/5)
CD2	0	5.239663	0.960167	0	1	0	0	0	Algorithm-Specific (1-2/5)
ACPP	-0.01994	5.226072	0.948975	1	0	0	0	0	Algorithm-Specific (1-2/5)
CD7	0	5.071843	0.898579	0	0	1	0	0	Algorithm-Specific (1-2/5)
FLT3LG	0	4.983934	1.25824	0	0	1	0	0	Algorithm-Specific (1-2/5)
FCMR	0	4.939348	1.073409	0	0	0	1	0	Algorithm-Specific (1-2/5)
IL11RA	-0.30811	4.937504	0.446399	1	0	0	0	0	Algorithm-Specific (1-2/5)

FCER1G	0	4.809588	2.048684	0	0	1	0	0	Algorithm-Specific (1-2/5)
CD247	0	4.723114	1.487911	0	0	1	0	0	Algorithm-Specific (1-2/5)
KLRK1	0	4.722287	0.542138	0	0	1	0	0	Algorithm-Specific (1-2/5)
LY9	0	4.585941	0.74014	0	0	1	0	0	Algorithm-Specific (1-2/5)
OCIAD2	0.060521	4.368813	1.276643	1	0	0	0	0	Algorithm-Specific (1-2/5)
SERPINA1	0	4.188975	1.21181	0	0	1	0	0	Algorithm-Specific (1-2/5)
TMIGD2	0.167702	3.970245	0.292945	1	0	0	0	0	Algorithm-Specific (1-2/5)
MAL	0	3.765701	0.71098	0	0	0	0	1	Algorithm-Specific (1-2/5)
ANO9	1.330315	3.731107	0.369844	1	0	0	0	0	Algorithm-Specific (1-2/5)
FCGR2A	-0.04759	3.678015	0.326117	1	0	0	0	0	Algorithm-Specific (1-2/5)
LIME1	0.463076	3.662858	0.662621	1	0	0	0	0	Algorithm-Specific (1-2/5)
TNFRSF25	0	3.612101	1.118021	1	0	0	0	0	Algorithm-Specific (1-2/5)
SKAP1	0.697835	3.490338	0.941951	1	0	0	0	0	Algorithm-Specific (1-2/5)
CD3E	0	3.341475	0.716604	0	0	0	0	1	Algorithm-Specific (1-2/5)
PVRIG	0.026099	3.322074	0.479981	1	0	0	0	0	Algorithm-Specific (1-2/5)
SHISAL2A	0.015744	2.907675	0.483018	1	0	0	0	0	Algorithm-Specific (1-2/5)

CSNK1E	-0.20843	2.725883	0.54941	1	0	0	0	0	Algorithm-Specific (1-2/5)
ADAMTS10	0	2.123675	0.221477	1	0	0	0	0	Algorithm-Specific (1-2/5)
LTBP3	-0.56153	2.118193	0.365857	1	0	0	0	0	Algorithm-Specific (1-2/5)
SBK1	0.316758	1.168719	0.460724	1	0	0	0	0	Algorithm-Specific (1-2/5)

Supplementary Table S5 QPCR primer sequence

Human		
Gene	Forward Sequence (5'-3')	Reverse Sequence (5'-3')
ADAP2	CGACTGGGCCTCTTACAAGC	ATGTCAGGGAAGTTACGGTGG
MERTK	CTCTGGCGTAGAGCTATCACT	AGGCTGGGTTGGTGAAAAACA
AXL	GTGGGCAACCCAGGGAAATATC	GTACTGTCCTGTTCGGAAAG
B-ACTIN	CCTGGCACCCAGCACAAT	GGGCCTGGACTCLTCAAC
Mouse		
Gene	Forward Sequence (5'-3')	Reverse Sequence (5'-3')
Adap2	gcctcttacaagctggggat	tcgtcccagaagtccagtct
Mertk	acgttggtggatacgtgcat	ctcttcccacttctcggcag
Axl	ttcaactgtgctacgtcccc	gggtccctctaggtaagcca
b-actin	gtgctatgttgctctagacttcg	atgccacaggattccatacc
